# Wheat Bran Polymer Scaffolds: Supporting Triple-Negative Breast Cancer Cell Growth and Development

**DOI:** 10.3390/bioengineering12060568

**Published:** 2025-05-26

**Authors:** Abulquasem Rayat Hossain, Md Sultan Mahmud, Kaydee Koistinen, George Davisson, Brooke Roeges, Hayle Boechler, Md Abdur Rahim Badsha, Md Rakib Hasan Khan, Michael Kjelland, Dorsa Fereydoonpour, Mohiuddin Quadir, Sanku Mallik, Khwaja Hossain

**Affiliations:** 1 Department of Pharmaceutical Sciences, North Dakota State University, Fargo, ND 58108, USA; abulquasem.hossain.1@ndsu.edu (A.R.H.);; 2 Spring Wheat Quality, North Dakota State University, Fargo, ND 58108, USA; mdsultan.mahmud@ndsu.edu; 3 Division of Science and Mathematics, Mayville State University, Mayville, ND 58257, USAmdabdurrahim.badsha@mayvillestate.edu (M.A.R.B.);; 4 College of Medicine, University of Saskatchewan, Saskatoon, SK S7N 5A5, Canada; 5 Biomedical Engineering, North Dakota State University, Fargo, ND 58108, USA

**Keywords:** arabinoxylan, breast cancer, hypoxia, MDA-MB-231 cell lines, scaffolds, spheroid, wheat bran

## Abstract

Arabinoxylans (AX) are functional biopolymers, the main non-starch polysaccharides in cereals and other plants. AX is composed of xylose and arabinose, and the ester-linkage of ferulic acid to arabinose confers its bioactive properties. The backbone of AX resembles that of glycosaminoglycans, a major component of the human extracellular matrix. This study explores the potential of wheat bran AX-based scaffolds as a novel platform for the growth and development of triple-negative breast cancer (TNBC) cells, an aggressive form of breast cancer. Importantly, patients face the worst prognosis due to the stemness of the TNBC cells and the formation of hypoxic cell clumps. Wheat bran constitutes 15–25% of the byproducts after milling and adds limited economic value. We have extracted AX from wheat bran (WBAX) and developed soft scaffolds with Na-alginate. The scaffolds were seeded with the triple-negative breast cancer cell line MDA-MB-231. Over 21 days, cell growth and development, cell migration within the hydrogels, and the formation of hypoxic regions within cell clumps were observed. These findings suggest that WBAX-based scaffolds provide a conducive environment for TNBC cell proliferation and development, offering a promising avenue for further research into cancer cell biology and potential therapeutic applications.

## 1. Introduction

In vitro research offers advantages over in vivo research, such as tight control of the chemical and physical environment, reduced costs, decreased animal use, and higher throughput. However, classic in vitro experiments fail to replicate the conditions of living organisms. Isolated primary cells grown in in vitro environments differ from those in living organisms, which limits the value of in vitro data for predicting in vivo behaviors. Current in vivo research involves testing living subjects, creating ethical dilemmas [1].

Cell-based processes often originate from experiments on rigid materials like polystyrene and glass that do not mimic physiological conditions. Cells in these environments tend to exhibit abnormal behaviors: flattened shapes, unusual polarization, altered responses to drugs, and a loss of their specialized characteristics. These culture systems are typically two-dimensional (2D) [2], whereas cells within the body receive signals from all dimensions, not just their bottom surface [3]. Culture systems are needed that more accurately replicate the biological conditions.

Biomaterials now provide greater complexity in cell culture by controlling mechanical, compositional, and structural cues to replicate native tissues [4]. Various systems have been developed, but hydrogel networks of water-swollen polymers have emerged as the most promising. Hydrogels mimic native extracellular matrices (ECMs), possess mechanical properties similar to soft tissues, and support cell adhesion and protein retention [5].

Biodegradable scaffolds can be implanted into defective tissue sites to support regeneration [6]. Cells infused onto scaffolds adhere in three dimensions (3D), proliferate, and secrete extracellular matrix [7,8]. Scaffolds are highly porous with an interconnected microarchitecture mimicking living organisms [9]. Scaffolds also provide pathways for nutrient transportation and cell signaling, promoting cell growth and tissue formation [10]. Arabinoxylan (AX) forms continuous and cohesive matrices, producing jelly-like films [11].

The biocompatibility of scaffold gels allows cells to adhere, function normally, migrate, and proliferate before creating new extracellular matrix [12]. Due to biodegradability, cells can produce extracellular matrix while degrading the scaffold. The balance between mechanical properties and porosity of the scaffold enables cell migration and proliferation. Well-prepared scaffolds contain interconnected pores for cellular penetration, nutrient diffusion, and excretion of waste products [13].

Hydrogels mimicking native extracellular matrix provide the mechanical environment for cellular responses and tissue creation in vitro [14]. Cells respond to mechanical signals, such as fluid shear, applied strains, and confinement, while also generating forces in their surroundings [15]. Stiffness is a key mechanical property of hydrogel, often used interchangeably with elasticity [16]. Physical interactions affect the hydrogel’s dynamic properties, such as stress relaxation with suitable stiffness. Alternating blocks of sugars with alginate interacting with divalent cations ensure biological applications [17], though stiffness changes with bivalent Ca+ concentration. Relative stiffness is quantified by the elastic modulus or a stress–strain tangent curve. Hydrogel stiffness can be tuned by altering the polymer concentration or cross-linking density, or both.

Hydrogels have proven useful in cell culture applications, revealing principles of cell behavior and offering tools for expanding various cell types in ways not achievable with conventional culture materials [18]. While both natural and synthetic polymer-based hydrogels have their merits, e.g., high mechanical strength, ease of processing, and stability, natural polymer-based hydrogels excel in bioactive properties, biocompatibility, and biodegradability [19]. Natural polymers serve a broader range of functions, facilitating better cell interactions and improving tissue performance [20]. Additionally, natural polymers more easily incorporate cell membrane receptors and peptide ligands to promote cell adhesion [21]. Another noteworthy feature of natural polymeric hydrogels is their capacity to retain cells and drugs, enabling controlled delivery, and they are suitable for modifications involving ligand attachment and crosslinking [22]. Natural polymer-based hydrogels have garnered significant attention in clinical applications due to their minimal provocation of inflammatory or immunological responses in host tissues.

The objective of this study was to develop and evaluate a novel, cost-effective hydrogel system using sodium alginate and wheat bran-derived AX for cultivating 3D triple-negative breast cancer spheroids. This hybrid gel system aims to provide a more physiologically relevant microenvironment for cancer cell growth while supporting high-content imaging assays for rapid assessment of cellular toxicity, uptake, and treatment effects. Specifically, MDA-MB-231 breast cancer cells were used, being the most frequently diagnosed cancer in women [23]. More than 40,000 women die from breast cancer in the US every year [24]. Triple-negative breast cancer cells (TNBC) are the most aggressive type and lack estrogen, progesterone, and HER2 receptors [25]. Researchers use established triple-negative breast cancer cell lines like MDA-MB-231 [26], which develop 3D spheroids [27], for studying cell–cell interactions and the tumor microenvironment. These cells require specialized conditions to study their behavior more accurately in the laboratory [28].

**Figure 1:**
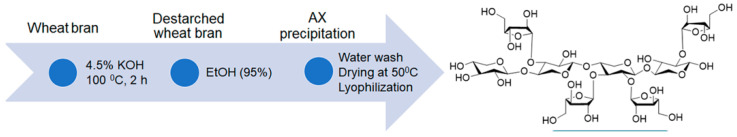
Extraction pathway of Arabinoxylan (AX) from wheat bran leading to AX [31].

AX was extracted from wheat bran collected from a North Dakota milling company. Wheat bran constitutes about 15–25% of milling byproducts but provides little profit due to transportation costs [29]. Wheat bran primarily comprises the outer kernel layers and is composed of insoluble AX, cellulose, starch, protein, β-glucan, and lignin [30]. The overall AX concentration ranges from 6.1% to 22.1% in bran and 1.4% to 2.8% in flour. Wheat bran AX constitutes approximately 29% of wheat’s total dietary fiber. Wheat bran is a valuable reservoir of hemicellulose compounds, with around 7.8% being water-soluble. AX, a polysaccharide, makes up 86.4% of the total water-soluble hemicellulose in wheat bran. AX, composed of arabinose and xylose pentose sugar units, displays a notably intricate molecular structure, characterized by a β-(1−4)-xylan backbone with α-(1−2) and α-(1−3) linked arabinose side chains (Figure 1) [31,32].

AX’s chemical structure offers a platform for delivering bioactive molecules due to its branching and multifunctional chemical groups. The self-assembling behavior of AX results from the hydrophobic interactions, van der Waals forces, and various intra- and intermolecular H-bonding among AX molecules. Previously, our group developed a facile and reproducible method to generate functional nanomaterials from wheat bran-derived AX and encapsulated the nanoparticle with nucleic acid and showed the potential of using AX for nucleic acid delivery. In this study, the spectroscopic characterization of AX, such as FTIR and ^1^H NMR, was conducted [31].

Sodium alginates from brown seaweed form hydrogels through ionic gelation with higher concentrations of Ca^2+^, Sr^2+^, or Ba^2+^ ions [33]. Research has shown that human intestinal organoids can be cultured in vitro and undergo epithelial differentiation in alginate hydrogels. Optimal cancer spheroid survival rates occurred in 1–2% alginate gels. The 0.5% alginate gel closely resembled those of commercial extracellular matrix (ECM) hydrogels [34]. To enhance cell adhesion and the growth of cancer spheroids, a hybrid alginate–AX hydrogel was prepared that is cost-effective compared to commercial ECM mixtures and compatible with high-content imaging assays for cellular toxicity assessment.

## 2. Materials and Methods

### 2.1. Extraction of AX from Wheat Bran

AX was extracted from wheat bran by a method developed by Lopez et al. with modifications following our previous work [31]. An amount of 40 g of wheat bran was mixed with 400 mL of 4.5% potassium hydroxide (KOH) solution and stirred at 100 °C for 2 h. The resultant slurry was then centrifuged for 20 min at 8885 g, and the supernatant was collected. Thereafter, two times the volume of 95% ethanol was added to the collected supernatant, and the suspension was stored at 4 °C for 16 h to ensure complete precipitation of AX. The precipitate was then collected by centrifugation (same speed and time as above) and was washed thoroughly with distilled water to remove ethanol and excess KOH. Subsequently, the AX was dried in a vacuum oven at 40 °C for 24 h and was lyophilized at −50 °C using a freeze-dryer (Modulyod, Thermo Electron Corporation, Waltham, MA, USA). The chemical structure and the extraction process of AX are shown in Figure 1.

### 2.2. Preparation for Stock Solutions

#### 2.2.1. Preparation of the 4% (*w*/*v*) Alginate Solution

Twenty-five milliliters of 4% (*w*/*v*) sodium alginate solution was prepared by dissolving 1 g of sodium alginate (Fisher Scientific, Hampton, NH, USA) in 25 mL of PBS (Fisher Scientific, USA) in a heat-resistant flask, stirring with magnetic stirrers. Sodium alginate was added slowly to avoid precipitating sodium alginate in PBS. The solution was incubated at 50 °C for 2 h to fully dissolve the sodium alginate. The solution was sterilized in an autoclave under 15 psi at 121 °C for 30 min.

#### 2.2.2. Preparation of the 2% (*w*/*v*) AX Solution

A volume of 25 mL of 2% (*w*/*v*) solution was prepared by dissolving 0.5 g of AX in 25 mL of ultrapure cell culture grade water (Fisher Scientific, USA) in a heat-resistant flask, and the AX was dissolved by continuously stirring the solution using a magnetic stirrer for 2 days. The AX solution was also autoclaved under the same conditions as the alginate solution.

### 2.3. Fabrication and Plating of Hydrogel

A working solution of hydrogel mix that contains 2% alginate and 1% AX (2:1) was prepared. A volume of 125 μL/well was prepared by mixing 62.5 μL of 4% (*w*/*v*) sodium alginate solution and 62.5 μL of 2% (*w*/*v*) AX solution. This mixing process was performed under the laminar flow hood (ESCO Class II BSC, Esco Technologies, Inc., St. Louis, MO, USA) with vigorous shaking until a uniform-colored mixture was achieved. Before adding the sodium alginate–AX mixture to the well of the 24-well cell culture plate (Fisher Scientific, USA), the wells were coated with poly-D-lysine to prevent the alginate gels from detaching from the cell culture plastics during 3D culture. A 0.5 mg/mL poly-D-lysine solution was prepared by dissolving the powder in ultrapure water, and the solution was sterilized by passing it through a 0.22 μm filter. Two-hundred-and-fifty microliters (250 μL) of the prepared poly-D-lysine was added to each well of a 24-well plate, and the plate was incubated for an hour under UV light. One-hundred-and-twenty-five microliters (125 μL) of the alginate–AX working solution were added to the well by making sure that the bottom surface of the well was covered. Polymerization of the hydrogel was achieved by adding 100 mM of sterile CaCl_2_ solution, which was prepared by dissolving 735 mg CaCl_2_ dihydrate in 50 mL ultrapure water and sterilized in a similar way to how the poly-D-lysine was sterilized. A volume of 100 μL of 100 mM CaCl_2_ sterile solution was carefully added to the hydrogel mix, and the solution was kept in a horizontal position undisturbed for 10 min at room temperature. Any unbound CaCl_2_ solution was removed carefully, and the hydrogel was washed with PBS.

### 2.4. Mechanical Properties of Hydrogels

Elastic Modulus: Nanoindentation tests were carried out to comprehend the mechanical properties of the prepared hydrogels. The elastic modulus of the hydrogel A: wheat bran AX (WBAX) and Na-alginate (SA) (1:2) was compared to B: sugar beet pulp arabinoxylan (SBAX) and SA (1:2), C: WBAX, SA, and collagen (C) (1:2:1), and D: SBAX, SA, and C (1:2:1). First, the gel samples were freeze-dried before placing into the indenter. Then, the samples were mounted on an iron base and loaded on the magnetic stage of the HYSITRON, T1970 triboindenter. Before creating an indent in the gel samples, the Berkovich diamond indenter was calibrated using a quartz specimen. The air indentation was performed to calibrate the transducer electrostatic force and check the transducer plate spacing. The contact area between the sample and the tip was used to determine the sample’s elastic modulus. The visible light and scanning probe microscopes connected to a triboindenter were used to identify the indentation. A minimum of three indentations were performed on each specimen, and the average value was reported. The load function for each indentation was chosen in such a way that the peak load value was 500 μN, while the loading time of 10 s was the same as the unloading time. The unloading curve slope was used to calculate the specimens’ modulus and hardness, and the integral software was used to establish the maximum depth to which the indenter could be forced.

Water-Holding Capacity (WHC): The Water-Holding Capacity is the maximum amount of water absorbed and retained by a material under certain conditions. It is usually expressed as the % of water retained per weight of dry materials, and is the most acceptable physicochemical property of the hydrogel for in vitro three-dimensional cell cultures [35,36,37,38,39]. The WHC of the hydrogel has been determined by following the method described by Shiwei [38]. The dry hydrogel was weighed as w_0_ and placed into a beaker containing 50 mL of distilled water. The gels were allowed to soak for 24 h. After 24 h at room temperature, the hydrogel was removed from the water and weighed as w_1_. Water-holding capacity (W_h_%) was determined using the following formula [35]

$$Wh\%=\frac{\left(w1-w0\right)}{w0}\times 100\%$$

where w_0_ = initial weight of the hydrogel in (g); w_1_ = final weight of the hydrogel after soaking in (g); and W_h_ = water holding capacity in %.

### 2.5. Cell Thawing, Cell Plating, Propagation, and Collection

Cryopreserved human breast cancer cell lines MDA-MB-231 (ATCC^®^) were thawed in a water bath at 37 °C. The MDA MB-231 cells were maintained in a monolayer within a T75 flask (Thermo Fisher Scientific, Waltham, MA, USA) in the DMEM-enriched medium (Cytiva, Marlborough, MA, USA). The cells were harvested once the cell culture flasks were 80% confluent. The flask was transferred into laminar flow and incubated at 37 °C after adding 6 mL of TrypLE, and the progress of the detachment of the cells was observed every 5 min until they were fully detached. After the cells were detached, an equal volume of media was added to quench the TypLE activity. The cell suspension was transferred into 50 mL centrifuge tubes (32.5 mL each) and centrifuged at 200× *g* for 10 min. The supernatant was removed, and the cells were resuspended in 15 mL of fresh media. Three hundred microliters (300 μL) of the MDA MB-231 suspension were seeded into each well of a 24-well plate (7500 cells/well) on the hydrogel. The plate was incubated at 37 °C, 5% CO_2_, for 21 days, with the culture medium refreshed every 4–5 days. A 40–1500× Inverted Phase-Contrast + Fluorescence Microscope fitted with 15× eyepieces (30 mm), UV-Filter (365 nm/450 nm), B1 (480 nm/535 nm), and G1 (560 nm/635 nm) filters, and a 6MP Extreme Low-light Camera (AmScope™, Irvine, CA, USA) were used to observe the progress of cell growth and development and image analysis.

### 2.6. Cell Staining

The cells in the 3D scaffold gels were stained to analyze the concentration of live and dead cells. SYBR green dye and red propidium iodide were added to the wells. The green dye penetrates all cells, living and dead, and the red dye only penetrates cells with membranes that are no longer intact. Therefore, the cells fluorescing green were living, and the cells fluorescing red were considered dead [20,40]. Live/dead cell staining was performed using SYBR^®^ Green I, nucleic acid gel stain, 10,000× concentrate in DMSO, Lot No. 24117W (Molecular Probes, Invitrogen™, Thermo Fisher Scientific, USA) and Propidium Iodide, Solution in Water, 1 mg/mL Lot: 19PO628, Mwt: 668.4 (Biotium, Inc., Fremont, CA, USA).

To determine the presence of hypoxia within the microenvironment, hydrogel cell cultures of MDA-MB-231 (ATCC^®^) were stained with green hypoxia dye, specifically Image-iT™ Green Hypoxia Reagent (5 µM), Lot No. I14833 (Thermo Fischer Scientific, USA). The dye does not become fluorescent until atmospheric oxygen levels are less than 5%, and it is irreversible even when oxygen levels are returned to normal. The Hypoxia dye was incubated with living cells for 1.5 h before being fixed with 4% paraformaldehyde for 15 min. Once imaged to capture the intensity of hypoxia, the fixed-hydrogel cell cultures were submerged with 0.1% Triton for 30 min, bovine serum albumin for blocking for 4 h, and fluorescent stain 4′,6-Diamidino-2-Phenylindole or DAPI (~1 ug/mL), Cat. No. 40011 (Biotium, Inc., USA) for 30 min to stain cell nuclei for identification. Images were captured with a Zeiss Airyscan Confocal Laser Scanning Microscope or AC-LSM (immunofluorescence, Zeiss Microscopy, North Dakota State University, Fargo, ND, USA).

## 3. Results and Discussion

### 3.1. Mechanical Properties of Hydrogels

**Figure 2:**
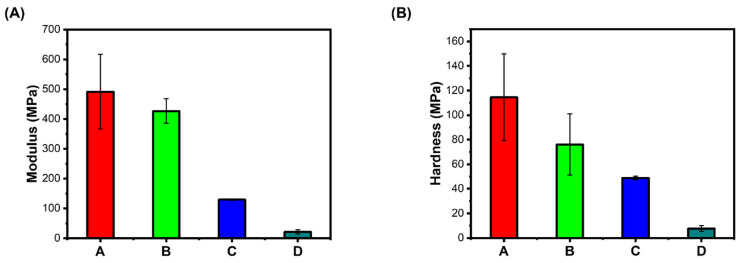
Gel mechanics (panel (**A**)) Modulus in MPa, (panel (**B**)) Hardness in MPa. A. (WBAX: SA 1:2); B. (SBAX: SA 1:2); C. (WBAX: SA: C 1:2:1); D. (SBAX: SA: C 1:2:1).

**Elastic Modulus**: Hydrogels mimicking the native extracellular matrix need to provide the cell’s local mechanical environment, guiding the encapsulated cells to cellular responses and creating tissues in vitro [14]. Cells respond to mechanical environmental signals, such as fluid shear, applied strains, and confinement. They also generate forces in their surroundings and assess the elastic and viscoelastic properties [15]. Stiffness is one of the important mechanical properties of hydrogel, often interchangeably used with the elasticity of hydrogel [16]. Physical interactions are important for hydrogel’s dynamic properties, such as stress relaxation with suitable stiffness. Alternating blocks of sugars with alginate interacting with divalent cations ensure the biological applications of hydrogel [17]; however, the stiffness of the hydrogel is often altered with changes in the concentration of bivalent Ca+. Relative stiffness is often quantified by elastic modulus or by the material stress–strain tangent curve at a given deformation. Whenever the stress–strain curve is linear, the elastic modulus is referred to as Young’s modulus. Depending on the chemistry and base material, the stiffness of hydrogel is often tuned over a wide range. The hydrogel with natural polymer has a lower bulk stiffness with a maximum elastic modulus of a few hundred Pa. The stiffness of hydrogel is often modulated by altering the polymer concentration, the density of cross-link, or both. Here, the modulus and hardness of our present hydrogel A. (WBAX and SA 1:2) were compared with hydrogels prepared with B. (SBAX and SA 1:2), C. (WBAX, SA, and C 1:2:1), and D. (SBAX, SA, and C 1:2:1), (1:2). In general, a wide range of modulus and hardness values was observed, which depends on the chemistry and base materials. Hydrogel stiffness is often modulated by altering either the polymer concentration, the density of cross-links, or both. Here, the cross-links were rendered by calcium chloride before seeding cells, but the mechanical properties were analyzed before adding calcium chloride, thus the variation in stiffness did not show the effect of cross-linking; however, a wider range of variation in A and B hydrogels compared to those of C and D was most likely because of collagen (Figure 2).

**Figure 3:**
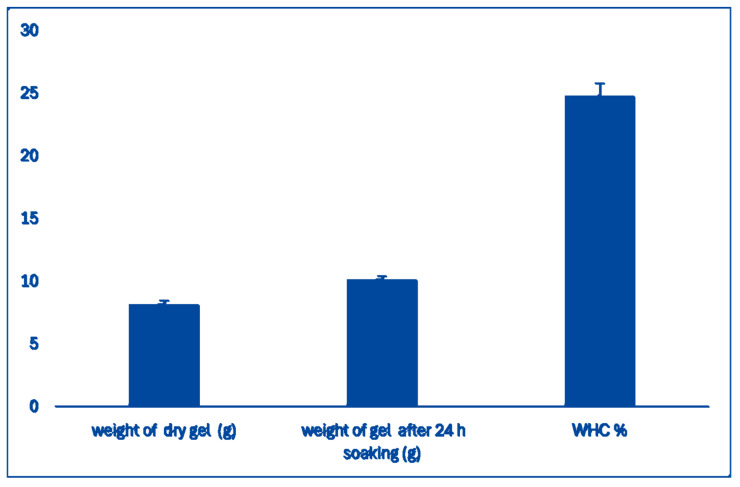
The water holding capacity of hydrogel. The columns represent dry weight, weight after soaking the dry components in water, and water holding capacity.

**Water-Holding Capacity (WHC)**: The WHC of the scaffold prepared with wheat bran AX and Na-Alginate was analyzed following the method described [39]. The hydrogel WHC was found to be 27% (Figure 3), which indicates that the hydrogel has enough water molecules for in vitro 3D cell culture [39].

### 3.2. Breast Cancer Cells in Hydrogel

The MDA-MB-231 cells grew in 2:1 sodium alginate to AX for approximately 21 days and were compared to their respective control environment on a 24-well cell culture plate. Cell growth in the control wells was extensive, with media needing to be replaced every three days to maintain cell growth. After five days of plating the cells, the cells were stained with SYBR green dye and red propidium iodide to identify live and dead cells.

**Figure 4:**
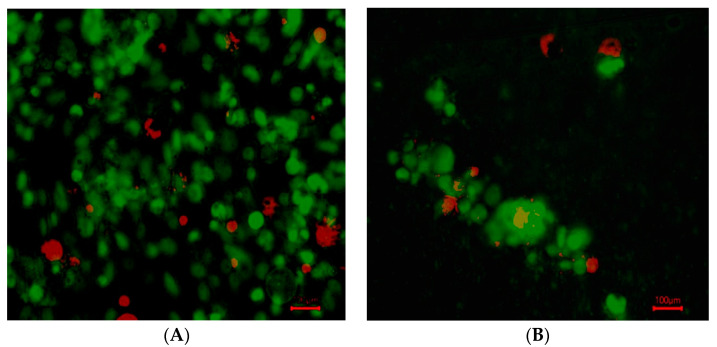
SYBR green-labeled MD MB 231 live cells and propidium-labeled dead cells after 5 days. (**A**). Live and dead MDA-MB-231 cells overlayed in control (no hydrogel). (**B**). Live and dead cells overlayed in hydrogel. The cells were of fibroblastic morphology, which is normal for that culture environment. (100 µm).

The cell growth in hydrogel wells was not as prevalent, with persisting cells found in small groups (10–20 µm in diameter) with a more rounded morphology (indicative of not attaching to a flat surface) (Figure 4).

**Figure 5:**
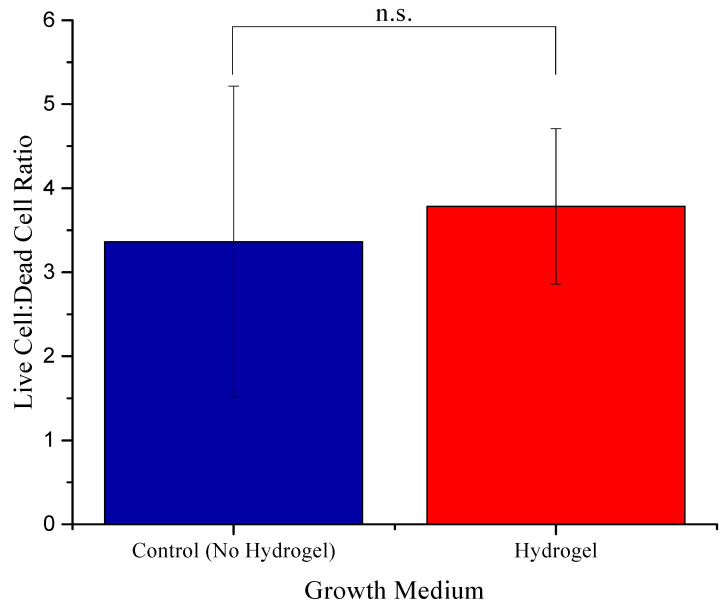
Comparison of the ratio of live and dead cells in the control (no hydrogel) and the hydrogel. n.s. is not significant.

Analysis was conducted through ImageJ software (V 1.49t) [41]. Images were first converted to 8-bit format and average intensity collected from the whole screenshot through the “Analysis” option (n = 4). The intensity of the live green stain and dead red stain from the corresponding screenshots was set up as a ratio, respectively. The “Bespeckle” function in Noise submenu of ImageJ replaces each pixel with the median value of its neighboring pixels, effectively eliminating background noise. To determine if the ratios were significantly different from one another, one-way ANOVA was conducted, with significance determined if *p* < 0.05. (Figure 5).

**Figure 6:**
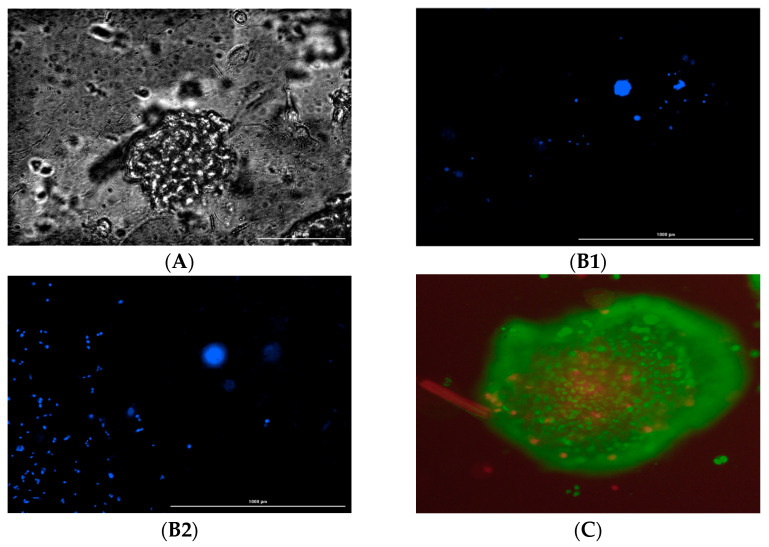
(**A**). Bright field image of MD MB 231. (**B**). Cells in different hydrogel planes. (**B1**). Focus on the monolayer of the bottom surface. (**B2**). Focus on approximately 370 microns above the bottom surface. (**C**). Hypoxia Region in the spheroid. Blue represents DAPI fluorescence, green represents SYBR green fluorescence, and red represents the propidium fluorescence.

No significant variation was observed in the ratio of live and dead cells in the control and hydrogel. Additionally, some cells grown in hydrogel were also found on the bottom surface of the plate with the fibroblastic morphology underneath the hydrogel, indicative of attachment to the plate surface. Although we did not measure the porosity or pore size of the hydrogel, focusing on different planes in the hydrogel ensured the presence of cancer cells in different layers of the hydrogel (Figure 6(B1,B2)).

**Figure 7:**
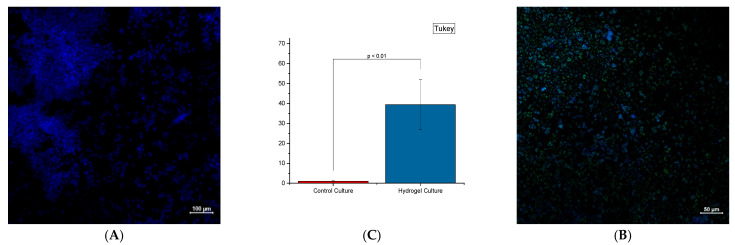
Confocal images of MDA-MB-231 cells grown in media (**left**) and a 1:2 WBAX to SA mixture (**right**) after 21 days. DAPI signaling (blue) indicates the nucleus of the cells, while Hypoxia dye (green) indicates the absence of oxygen. Objective data were collected and compared with each other (n = 3). (**A**). MDA-MB-231 cells grown in control well (media only) (**B**). MDA-MB-231 cells grown in experimental well WBAX and SA (2:1) (**C**). Significant difference *p* < 0.1 in hypoxia-specific dye intensity.

The fluorescence dye in triple-negative breast cancer cells in three dimensions showed characteristic features, such as hypoxic niches and the associated cancer stem cells (Figure 7). Confocal Immunofluorescence imaging with the hypoxia dye has shown a significant presence (*p* < 0.01) of hypoxia with cells grown in and underneath the hydrogel compared to their respective control wells (significance determined via Student’s *t*-test).

## 4. Conclusions

As for the in vitro component concerning AX, the breast cancer MDA-MB-231 cells were cultured successfully in 2D and 3D conditions. However, there was a clear difference in morphology between the control and AX treatment groups, with a greater degree of clumping of cells (characteristic of early-stage tumor spheroid formation) in the AX treatment group. The scaffolds, prepared with wheat bran AX mixed with Na-alginate, showed sustainable growth and development of cancer cells (up to 21 days). This natural polymer-based scaffold may have excellent biocompatibility and tunable mechanical properties. However, further studies on cell growth and development are important.

Cell growth of MDA-MB-231 in hydrogels persisted for up to three weeks with continuous cellular respiration (as indicated by a change in phenol red color of DMEM cell culture media). Morphology change was shown due to the absence of a fixed surface for cellular attachment, but this did not deter cell growth. Additionally, the WBAX and sodium alginate hydrogel induced a hypoxic microenvironment for the cells to grow, which closely mimics solid tumor conditions compared to conventional cell culture circumstances. More studies to assess phenotypic alterations and cancer stemness will need to be conducted to determine whether or not this hydrogel formulation induces similar changes seen in cells grown in solid tumor physiological settings. We have developed more hydrogel systems using AX from another agricultural byproduct, sugar beet pulp, and incorporated a natural protein source such as collagen.

## Data Availability

The original contributions presented in the study are included in the article, further inquiries can be directed to the corresponding author.
